# The effects of weight self-stigma on psychological distress in adolescents: the chain-mediated roles of fear of negative appearance evaluation and social appearance anxiety

**DOI:** 10.3389/fpsyg.2025.1619694

**Published:** 2025-09-18

**Authors:** Changhao Tang, Xinmiao Zhang, Chuncen Zhou, Kai Li, Yujun Cai

**Affiliations:** ^1^School of Physical Education, Shanghai University of Sport, Shanghai, China; ^2^College of Sports Industry and Leisure, Nanjing Sport Institute, Nanjing, China

**Keywords:** weight self-stigma, psychological distress, fear of negative appearance evaluation, social appearance anxiety, adolescents

## Abstract

**Purpose:**

This study aimed to investigate the effects of weight self-stigma on psychological distress in adolescents and to examine the chain-mediating roles of fear of negative appearance evaluation and social appearance anxiety.

**Patients and methods:**

A multistage sampling method was used to conduct a questionnaire survey of 2,076 adolescents in Changsha, Zhuzhou, and Yueyang in Hunan Province. The Weight Self-Stigma Questionnaire, the Fear of Negative Appearance Evaluation Scale, the Social Appearance Anxiety Scale, and the Depression, Anxiety, and Stress Scale-21 were used in the investigation. The collected data underwent descriptive statistical analyses, correlation analyses, and chain mediation model tests.

**Results:**

A significant positive correlation was observed between adolescent weight self-stigma and fear of negative appearance evaluation, social appearance anxiety, and psychological distress (*p* < 0.01). Weight self-stigma was found to affect psychological distress in adolescents (effect value = 0.087) directly and also exert an indirect effect through the mediating roles of fear of negative appearance evaluation (effect size = 0.062) and social appearance anxiety (effect size = 0.061), as well as the chain mediating effect of these two indirectly affects psychological distress (effect size = 0.137).

**Conclusion:**

Weight self-stigma has a direct and positive impact on adolescent psychological distress. Fear of negative appearance evaluation and social appearance anxiety serve as mediators in this relationship. Meanwhile, fear of negative appearance evaluation and social appearance anxiety could also chain-mediate the relationship between adolescent weight self-stigma and psychological distress. It is necessary to develop effective intervention strategies to enhance physical activity and dietary management, thereby reducing weight self-stigma and promoting healthy physical and mental development in adolescents.

## 1 Introduction

Overweight and obesity are global public health problems. According to World Health Organization (WHO) data, the total number of children with obesity, adolescents, and adults worldwide has surpassed 1 billion. By 2022, 159 million children and adolescents were affected by obesity, with an obesity rate four times higher than in 1990 (Phelps et al., 2024). Compared with their normal-weight peers, overweight or adolescents with obesity not only face health crises but also are at risk of social isolation (Puhl and King, 2013; Mannan et al., 2016; Pont et al., 2017), interpersonal alienation, and both verbal and cyberattacks, as well as a higher risk of physical injury (Waasdorp et al., 2018). Additionally, overweight and adolescents with obesity often experience weight-related stigma (Andreyeva et al., 2008), including public criticism, and subtle or indirect insults. Weight stigma refers to negative attitudes, opinions, and discriminatory behaviors toward individuals with obesity. Depending on its manifestation, its subordinate concepts include weight self-stigma (self-blame, self-devaluation), conscious stigma (fear of being discriminated against by others because of having real or imagined characteristics), and expressive stigma (the process of being blatantly discriminated against in reality) (Bharat et al., 2001). Among them, weight self-stigma refers to the internalization of negative societal evaluations and prejudices regarding body weight. Individuals may perceive their weight or body size as inconsistent with dominant societal esthetic standards (e.g., overweight or obesity), leading to self-devaluation, shame, or self-criticism (Lillis et al., 2010). Weight self-stigma affects individuals across different weight categories, but it is particularly pronounced among overweight and individuals with obesity (Himmelstein et al., 2017). Adolescents are commonly subjected to teasing, taunting, and bullying because of their weight (Munir and Dawood, 2021), which negatively affects their psychological well-being, leading to higher levels of anxiety, lower levels of self-esteem, and greater levels of stress (Jackson et al., 2015; Papadopoulos and Brennan, 2015; Wu and Berry, 2018).

In recent years, the international community has increasingly focused on the issue of weight stigma. In 2020, a multi-disciplinary group of international experts, including representatives of ten scientific organizations, released the Joint International Consensus Statement for Ending Stigma of Obesity, emphasizing the far-reaching impacts of obesity stigma on both individuals and societies and calling for action to reduce such stigma (Rubino et al., 2020). Certainly, this phenomenon has gained the attention of scholars worldwide, with a range of studies being conducted around this topic. However, current research on weight self-stigma focuses more on college students and adult women with obesity (Forbes and Donovan, 2019; Prunty et al., 2020; Schram et al., 2024), while adolescents remain understudied. Adolescents are in the sensitive period of body image, and studying their unique psychological response mechanisms (e.g., high sensitivity to peer evaluations) have greater ecological validity and relevance. Additionally, the mediating variables in existing studies are relatively homogeneous (Curll and Brown, 2020), mainly focusing on food addiction, eating behaviors, and quality of life (Farhangi et al., 2017; Ahorsu et al., 2020), with limited exploration of other related variables and deeper mechanisms of influence.(Dapeng and Wen, 2020; Wen et al., 2023a; Wen et al., 2023b; Wen et al., 2024a). Therefore, the present study aims to enrich the research related to the mechanisms of the relationship between weight self-stigma and psychological distress in an adolescent population and to explore the chain-mediating roles of fear of negative appearance evaluation and social appearance anxiety in this relationship.

### 1.1 Weight self-stigma and psychological distress

Psychological Distress is a common mental health problem (Doherty et al., 2008; Drapeau et al., 2012; Marchand et al., 2012) and refers to a nonspecific, persistent negative psychological state experienced by individuals at the emotional, cognitive, and behavioral levels. It is characterized by subjective feelings of distress, imbalance, or difficulties in adjustment that do not yet meet the diagnostic criteria for a clinical psychological disorder (Kessler et al., 2003). The main manifestations of psychological distress are depression and anxiety (Ridner, 2004; Doran, 2011; Drapeau et al., 2012), and these symptoms usually co-occur (Van Oppen et al., 2007) with common somatic illnesses (Kroenke, 2003; Löwe et al., 2008; Haftgoli et al., 2010; Kroenke et al., 2013) and a variety of chronic diseases (Evans et al., 2005), as well as medically unexplained syndromes (Henningsen et al., 2003; Fagring et al., 2008). Survey data indicate that 32.9% of adolescents across four Asian countries have experienced psychological distress (Lee et al., 2019). Studies suggest that psychological distress is more prevalent among adolescents than in the general population and may lead to declines in academic performance and health among adolescents, with negative effects potentially into adulthood (Gebremedhin et al., 2020). Given the high prevalence and severity of psychological distress among adolescents, it is essential to identify the risk factors associated with psychological distress and explore effective strategies for improvement. A range of factors that induce psychological distress such as family relationships (Marchand et al., 2005), work stress (Faragher et al., 2005; Geisler et al., 2019), social support (Kendler et al., 2005), weight stigma and discrimination, loneliness (Beutel et al., 2017; Liu et al., 2020), and unhealthy lifestyles (e.g., smoking and alcohol abuse) (Grant et al., 2004; Berg et al., 2019; Ranjit et al., 2019; Li et al., 2020) have been identified.

Weight self-stigma, as a form of discrimination and prejudice, has negatively impacted adolescents’ physical and mental health to varying degrees. Research indicates that when individuals exhibit higher levels of weight self-stigma, the negative psychological impact on them is stronger (Latner et al., 2014; Papadopoulos and Brennan, 2015). Furthermore, weight self-stigma experiences have been shown to predict a range of adverse psychological outcomes such as depression, anxiety, psychological distress, body dissatisfaction, and low self-esteem (Stunkard et al., 2003; Annis et al., 2004; Vartanian and Smyth, 2013; Emmer et al., 2020). There may be an association between weight self-stigma and psychological distress (Wu and Berry, 2018; Emmer et al., 2020), and a growing body of evidence suggests that weight self-stigma is significantly and positively associated with depression (Özdemir and Türkben, 2023), anxiety, and stress (Ali et al., 2024), which are important indicators of psychological distress (O'Brien et al., 2016; Hayward et al., 2018). Accordingly, this study proposed the following research hypothesis H1: Adolescent weight self-stigma positively predicts psychological distress.

### 1.2 Mediating role of fear of negative appearance evaluation

Weight Self-stigma can be understood as the process by which individuals who are overweight or obese interact with the contemporary social and cultural ideal of beauty, accumulating a certain amount of negative cognitive experience about their bodies and appearance, and forming a relatively stable cognitive structure that negatively evaluates their appearance (Tomiyama, 2014). Appearance is the primary expression of individual charisma in interpersonal communication within contemporary society. It represents the external presentation of an individual, encompassing aspects such as appearance, body shape, and temperament. Moreover, appearance functions as a social construct, which is the first impression of others’ perception of an individual, is often evaluated by others and brings a corresponding emotional experience to the evaluated person (Wen et al., 2024b). In reality, there exists a generalized fear of negative evaluation, within which fear of negative appearance evaluation is a unique component related to body image (Lundgren et al., 2004). The fear of negative appearance evaluation refers to the emotions of worry and distress experienced by individuals as a result of negative, negative appearance evaluations given by others (Thomas et al., 1998). One of the first scholars to research weight stigma and appearance evaluation was Goffman (1963), who argued that body stigma is due to body defects or body social deviance. Weight self-stigma demonstrates appearance self-devaluation, body dissatisfaction, and weight concerns (Almenara et al., 2017) and will result in producing higher levels of negative appearance evaluation experiences (Stevens et al., 2018).

People’s evaluations of and concerns about others can affect an individual’s psychological functioning (Rapee and Heimberg, 1997; Bilge and Kelecioğlu, 2008). Therefore, fear of negative appearance evaluation is an important predictor of psychological functioning and psychological adjustment, and high levels of negative evaluation fear adversely affect an individual’s mental health (Button et al., 2015). Studies have shown that negative appraisal fear is significantly and positively associated with various psychological problems and that individuals with lower levels of this fear exhibit reduced anxiety and milder depressive symptoms (Kornienko and Santos, 2014). On the other hand, individuals with higher levels of negative appearance evaluation fear tend to be more vulnerable, they fear losing social appreciation and show higher levels of psychological distress than those with lower levels of negative appraisal fear (Nonterah et al., 2015). Research suggests that weight self-stigma may positively predict negative appearance evaluation fear, which in turn may positively predict psychological distress. Accordingly, this study proposed the following research hypothesis H2: Fear of negative appearance evaluation mediates the relationship between weight self-stigma and psychological distress in adolescents.

### 1.3 Mediating role of social appearance anxiety

Social anxiety refers to the fear of socializing that may lead to embarrassment or humiliation (Edition, 1980). The anxiety that arises when an individual fears negative evaluation by others based on their overall appearance (including shape and looks) is termed Social Appearance Anxiety (SAA) (Hart et al., 2008). Social appearance anxiety is a composite concept that combines social anxiety with negative body image, describing the extent of social anxiety an individual experiences regarding their overall appearance (including but not limited to body shape) (Hart et al., 2008). It has been suggested that the fear of being negatively judged in social interactions is a key feature of social appearance anxiety (Horenstein et al., 2021). Social appearance anxiety has been associated not only with teasing and bullying in general (Roth et al., 2002; McCabe et al., 2003; Erath et al., 2008), but also with experiences of weight stigma (Annis et al., 2004). Furthermore, the level of social appearance anxiety reflects individuals’ concerns regarding weight and body size (Hart et al., 2008), and individuals with higher levels of weight self-stigma are more likely to induce social appearance anxiety.

Individuals with high levels of social appearance anxiety have a strong sense of appearance inadequacy and are extremely sensitive to the measurements and evaluations of others, and internalizing these evaluations can harm an individual’s psychological well-being. It has been suggested that individuals with higher levels of social appearance anxiety lack confidence in themselves, show weaker interpersonal skills in social situations, and display pessimistic attitudes toward social interaction, leading to a higher probability of developing psychological disorders such as depression (Shepherd et al., 2019; Hussain et al., 2023). Other studies have shown that levels of social appearance anxiety can also influence an individual’s emotional state, with higher levels of appearance anxiety tending to be accompanied by less positive and more negative emotions (Harper and Tiggemann, 2008). In summary, weight self-stigma may predict social appearance anxiety, which in turn may predict psychological distress. Accordingly, the present study proposes the following hypothesis H3: Social appearance anxiety mediates the relationship between weight self-stigma and psychological distress in adolescents.

### 1.4 Chain mediation of fear of negative appearance evaluation and social appearance anxiety

Fear of Negative Appearance Evaluation (FNAE) and Social Appearance Anxiety (SAA) are conceptually similar, but they are also fundamentally different. FNAE is used to describe an anticipatory concern that individuals may be subject to negative appraisals of their physical appearance by others, with potential areas of concern including body size, facial features and clothing (Lundgren et al., 2004), whereas SAA is a comprehensive emotional response to the fear of being negatively appraised for one’s overall appearance in social settings, often accompanied by behavioral avoidance tendencies (Hart et al., 2008). FNAE focuses on the cognitive level of fear and is more focused on the degree of worry, whereas SAA encompasses emotional experiences and behavioral responses and focuses on the situational response. FNAE is often used as an antecedent variable for SAA, for example, FNAE is the fear of being criticized and SAA is the anxiety response due to the fear of being criticized. Anxiety response that arises. In addition, FNAE and SAA are triggered in different contexts, with FNAE being triggered in a generalized manner without actual social interaction, whereas SAA needs to be triggered in a specific social situation. Fear of negative appraisal has been recognized as a cognitive and emotional risk factor for social anxiety (Haikal and Hong, 2010), with a strong association between the two (Zhong and Zhang, 2011; Zhao and Dai, 2016). A significant part of social anxiety stems from fear of negative evaluations and excessive self-focus (Heimberg, 1995; Rapee and Heimberg, 1997). For example, a study on “social appearance anxiety in patients with eating disorders” found that social appearance anxiety was strongly associated with threatening information in the situation, which was mainly derived from negative evaluations related to appearance (Claes et al., 2012). Other studies have shown that when individuals’ social appearance anxiety is aroused, they subsequently report negative psychological traits related to their appearance, such as negative appearance appraisal fears (Coles et al., 2001). However, previous studies have mainly focused on either exploring individuals’ negative appraisal fears resulting from social anxiety (Koskina et al., 2011), or the mediating role of negative appraisal fears in the relationship between appearance and social anxiety (Li et al., 2023). This raises the question: does a similar relationship exist between fear of negative appearance evaluation and social appearance anxiety?

The social identity threat model of stigma explains that when people are in stigmatized situations, such as negative appearance evaluations based on body weight, involuntary stress responses such as social appearance anxiety will be induced (Major and O’brien, 2005; Almenara et al., 2017). The higher an individual’s perceived BMI (Ahadzadeh et al., 2018), the more dissatisfied they are with their appearance (Wan et al., 2024), and the more likely it is that this will lead to their fear of being negatively evaluated by others because of their appearance in social situations (Wan et al., 2024). Individuals who fear being judged based on their appearance often avoid unnecessary social interactions to escape negative appearance evaluations and criticisms that cause them distress (Rapee and Heimberg, 1997; Çetin et al., 2010) seeking a greater sense of security (Uğur et al., 2021). In summary, this evidence suggests that fear of negative appearance evaluation and social appearance anxiety may play some potential role in the relationship between weight self-stigma and psychological distress. Based on this, the present study proposes the following research hypothesis H4: Fear of negative appearance evaluation and social appearance anxiety play a chain mediating role in the relationship between weight self-stigma and psychological distress in adolescents.

**Figure 1:**
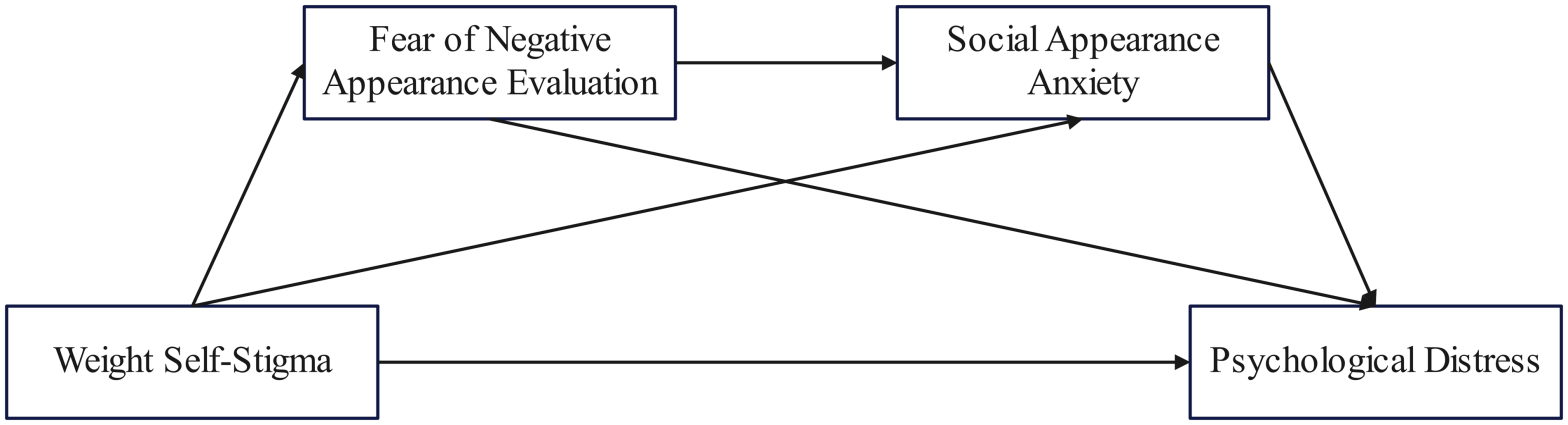
Chain mediation hypothesis model.

Collectively, this study proposes four research hypotheses and constructs a chain mediation model, as shown in Figure 1:

*Hypothesis 1* (H1): Adolescent weight self-stigma positively predicts psychological distress.

*Hypothesis 2* (H2): Fear of negative appearance evaluation mediates the relationship between weight self-stigma and psychological distress in adolescents.

*Hypothesis 3* (H3): Social appearance anxiety mediates the relationship between weight self-stigma and psychological distress in adolescents.

*Hypothesis 4* (H4): Fear of negative appearance evaluation and social appearance anxiety play a chain mediating role in the relationship between weight self-stigma and psychological distress in adolescents.

## 2 Materials and methods

### 2.1 Participant and procedure

This study used a multistage sampling method and chose a two-stage sampling strategy. First, Changsha City, Zhuzhou City and Yueyang City in Hunan Province were selected as the survey area according to geographic divisions; then, six schools were selected for the survey in the survey area, including two each of primary schools, junior high schools and senior high schools, and the test was administered collectively on a class-by-class basis. The study was reviewed and approved by the Ethics Committee of Shanghai Sport University. Informed consent was obtained from all participants, and the data collection process was conducted anonymously to protect participants’ privacy and mitigate potential social desirability bias. The questionnaires were completed in class under the guidance of the instructor and were uniformly distributed and collected. A total of 2,287 questionnaires were distributed, and 2,216 questionnaires were returned, which is a 96.90% response rate. After excluding incomplete or invalid questionnaires with missing answers or patterned responses, 2076 valid questionnaires remained, yielding a validity rate of 93.68%.

The age of the participants ranged from 12 to 18 years old, with 350 at 12 years old, 443 at 13 years old, 249 at 14 years old, 270 at 15 years old, 254 at 16 years old, 245 at 17 years old, and 265 at 18 years old. Of these, 1,102 were boys and 974 were girls. Detailed demographic information is given in Table 1.

**Table 1:** Characteristics of the study participants (*n* = 2,076).

Demographic variables	*n* (%)
Age	
12	350 (16.9)
13	443 (21.3)
14	249 (12.0)
15	270 (13.0)
16	254 (12.2)
17	245 (11.8)
18	265 (12.8)
Gender	
Males	1,102 (53.1)
Females	974 (46.9)
Weight status	
Low weight	845 (40.7)
Normal weight	1,022 (49.2)
Overweight	144 (6.9)
Obesity	65 (3.1)
Address	
Urban	386 (18.6)
Town	1,081 (52.1)
Rural	609 (29.3)
Only child status	
Only child	432 (20.8)
Not an only child	1,644 (79.2)
Household income	
Low	310 (14.9)
Middle	1,415 (68.2)
High	351 (16.9)

### 2.2 Measures

The Weight Self-Stigma Questionnaire (WSSQ) developed by Lillis et al. (2010) was used to measure weight self-stigma in adolescents. The questionnaire consists of two dimensions, self-devaluation (six items) and fear of enacted stigma (six items), and was used to assess the degree of self-devaluation resulting from adolescents’ internalization of others’ negative weight evaluations, with a total of 12 items scored on a Likert 5-point scale ranging from ‘1-Completely disagree’ to ‘5-Completely agree’, with higher scores indicating higher levels of weight self-stigma. The questionnaire had Cronbach’s alpha of 0.846 in this study.

The Fear of Negative Appearance Evaluation Scale (FNAES) developed by Lundgren et al. (2004) was used to measure adolescents’ fear of negative appearance evaluation. The scale consists of six items to evaluate the frequency of adolescents’ appearance being evaluated negatively by others and resulting in worry. A Likert 5-point scale was used, ranging from ‘1-Not at all’ to ‘5-Always,’ with higher scores indicating greater fear of negative appearance evaluation. The scale demonstrated high internal consistency, with a Cronbach’s alpha of 0.894 in this study.

The Social Appearance Anxiety Scale (SAAS) developed by Hart et al. (2008) was used to measure social appearance anxiety in adolescents. The scale has demonstrated good reliability and validity in studies of Chinese student populations (Li, 2020). There are 16 items, all of which are rated on a Likert 5-point scale ranging from ‘1-Very inconsistent’ to ‘5-Very consistent’. Higher scores indicate higher levels of social appearance anxiety, and item 1 was reverse-scored. The scale had a Cronbach’s alpha of 0.920 in this study.

The Depression, Anxiety, and Stress Scale-21 (DASS-21) developed by Lovibond and Lovibond (1995) was used to assess Psychological Distress in adolescents. The scale consists of 21 items divided into three dimensions including depression, anxiety, and stress, each containing seven items. A Likert 4-point scale was used, ranging from ‘0 - Does not apply to me at all’ to ‘3 - Applies to me very much or most of the time’. The psychological distress score is derived from the sum of all items, with total scores ranging from 0 to 63, with higher scores indicating more severe psychological distress. The scale has a Cronbach’s alpha of 0.907 in this study.

### 2.3 Statistical analysis

In this study, SPSS 23.0 was used for scale data reliability analysis, descriptive statistics, reliability analysis, and correlation analysis. Common method bias was tested using the Harman one-way test and Pearson correlation analysis was used to describe the correlation coefficients between variables. Then, the SPSS plug-in PROCESS provided by Hayes (2013) was used and model 6 was selected based on the template with a sampling number of 5,000. The chain-mediated effects of fear of negative appearance evaluation and social appearance anxiety were tested using demographic factors such as age, gender, weight status, place of residence, being an only child, and household income as control variables. A significant effect was indicated when the 95% Bootstrap confidence interval did not contain zero (Hayes, 2013).

## 3 Results

### 3.1 Common method biases analyses

The data for this study were derived from subjects’ self-reports, which may have resulted in common method bias. After the possible common method bias was controlled procedurally by anonymous completion and reverse scoring of some items, the common method bias was examined using the Harman one-way test. The results showed that there were a total of nine factors with eigenvalues values greater than 1 in this study and that the explained variance of the first factor was 28.97%, which was lower than the critical indicator of 40% (Dandan and Zhonglin, 2020), indicating that there was no obvious common method bias in the data of this study.

### 3.2 Descriptive statistics and correlation analysis

Descriptive statistics and correlation analyses were conducted on weight self-stigma, fear of negative appearance evaluation, social appearance anxiety, and psychological distress. The results showed that there was a significant positive correlation between weight self-stigma, fear of negative appearance evaluation, social appearance anxiety, and psychological distress (*p* < 0.01). The details are shown in Table 2.

**Table 2:** Descriptive statistics and correlation analysis between variables (*n* = 2,076).

Variables	M	SD	1	2	3	4
1. Weight self-stigma	23.23	7.54	1			
2. Fear of negative appearance evaluation	15.14	6.35	0.490**	1		
3. Social appearance anxiety	30.66	11.28	0.436**	0.693**	1	
4. Psychological distress	13.12	9.72	0.346**	0.497**	0.592**	1

**p < 0.01; M, mean; SD, standard deviation.

### 3.3 Chain-mediated role of fear of negative appearance evaluation and social appearance

**Figure 2:**
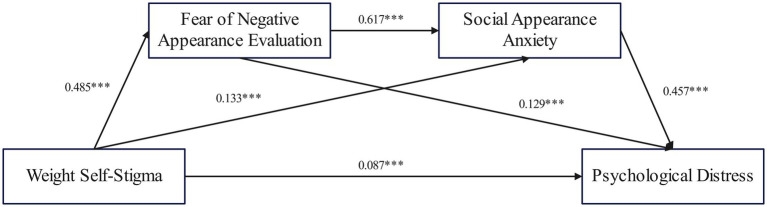
Intermediation effect diagram.

The results of the correlation analyses met the statistical requirements for further mediation effect tests involving fear of negative appearance evaluation and social appearance anxiety (Zhonglin and Baojuan, 2014). The mediated effects test based on the bootstrap sampling method was performed using the SPSS plug-in PROCESS provided by Hayes (2013), specifically applying Model 6 for model testing with a sample size of 5,000. Weight self-stigma was analyzed as the independent variable, psychological distress as the dependent variable, fear of negative appearance evaluation and social appearance anxiety as the mediator variables, and demographic factors such as age, gender, weight status, place of residence, being an only child, and household income as the control variables. The results showed that: weight self-stigma significantly and positively predicted psychological distress (*β* = 0.087, *p* < 0.001), weight self-stigma significantly and positively predicted fear of negative appearance evaluation (*β* = 0.485, *p* < 0.001), weight self-stigma significantly and positively predicted social appearance anxiety (*β* = 0.133, *p* < 0.001), and fear of negative appearance evaluation significantly and positively predicted psychological distress (*β* = 0.129, *p* < 0.001), fear of negative appearance evaluation significantly positively predicted social appearance anxiety (*β* = 0.617, *p* < 0.001), and social appearance anxiety significantly positively predicted psychological distress (*β* = 0.457, *p* < 0.001). The specific paths are shown in Figure 2.

The results of the mediation effect analysis showed that the indirect effect with fear of negative appearance evaluation as the mediating variable was 0.062 (95% CI = [0.035, 0.092]), accounting for 17.86% of the total effect. The indirect effect with social appearance anxiety as the mediating variable was 0.061 (95% CI = [0.041, 0.081]), contributing to 17.59% of the total effect. The combined indirect effect with fear of negative appearance evaluation and social appearance anxiety as mediating variables was 0.137 (95% CI = [0.116, 0.159]), representing 39.48% of the total effect, which indicates the establishment of a chained mediating effect for fear of negative appearance evaluation and social appearance anxiety. Overall, the total of all indirect effects was 0.260 (95% CI = [0.229, 0.292]) accounting for 74.93% of the total effect (Table 3).

**Table 3:** Bootstrap analysis of significance tests for mediating effects.

Pathway relationships	*β*	SE	95% Confidence Interval		Effect
			LLCI	ULCI	
WSS → PD	0.087	0.021	0.046	0.128	25.07%
WSS → FNAE→PD	0.062	0.014	0.035	0.092	17.86%
WSS → SAA → PD	0.061	0.011	0.041	0.081	17.59%
WSS → FNAE → SAA → PD	0.137	0.011	0.116	0.159	39.48%
Total indirect effect	0.260	0.016	0.229	0.292	74.93%
Total effect	0.347	0.021	0.306	0.389	100.00%

WSS, weight self-stigma; FNAE, fear of negative appearance evaluation; SAA, social appearance anxiety; PD, psychological distress.

## 4 Discussion

### 4.1 The effects of adolescent weight self-stigma on psychological distress

The results of this study indicated that adolescents had a significant positive effect on psychological distress, supporting Hypothesis H1. The relationship between adolescent weight self-stigma and psychological distress has been confirmed by several studies (O'Brien et al., 2016; Hayward et al., 2018; Chan et al., 2019; Lin et al., 2019; Ahorsu et al., 2020), and the present study further supports these ideas. The relationship between weight self-stigma and psychological distress can be explained through the developmental mechanism of weight self-stigma, adolescence is a dynamic period influenced by diverse emotional, social, and neurobiological factors (Hazen et al., 2008), when adolescents in this complex period are subjected to negative evaluations of their weight by teachers, peers, parents, etc., and perceive that they are the ones who fit into such a weight norm, adolescents accept negative evaluations of their own weight held by those around them, which can lead to self-defeating thoughts or beliefs, weight self-stigma exacerbates self-depreciation and self-denial in adolescents (Myre et al., 2021), such as self-attribution of ‘obesity’ as ‘incompetence’, decreased self-esteem, depression, and generalized anxiety, leading to psychological distress. According to the stress-coping model, the social discrimination and prejudice associated with weight self-stigma exceed adolescents’ coping capacity, causing them to perceive weight self-stigma as a significant stressful life event (Nichelson, 2020), and long-term psychological stress (or distress) can lead to a variety of unhealthy outcomes (Rog et al., 2024). Additionally, since adolescents’ physical and mental development is still immature, they may experience greater distress due to weight self-stigma. Combined with academic pressures and high expectations from parents and teachers, adolescents become more prone to psychological distress problems (Costello et al., 2011; Thapar et al., 2012; Rog et al., 2024).

### 4.2 Mediating role of fear of negative appearance evaluation

The results of the study indicated that fear of negative appearance evaluation partially mediated the relationship between weight self-stigma and psychological distress in adolescents, supporting Hypothesis H2. This finding aligns with previous research stating that ‘weight self-stigmatizes produce higher levels of negative appearance evaluation experiences’ (Stevens et al., 2018) and ‘individuals with higher levels of fear of negative evaluation exhibit higher levels of psychological distress’ (Nonterah et al., 2015). Weight self-stigma leads to self-devaluation regarding one’s appearance, increasing shame, guilt, and distress due to negative appearance evaluations from others. This, in turn, fosters negative body-related self-consciousness and emotional distress (Lucibello et al., 2021), which ultimately contributes to psychological distress. An experiment with college students showed that media images presenting weight stigma triggered fear of negative appearance evaluation (Boersma and Jarry, 2013), further supporting that weight self-stigma is related to fear of negative appearance evaluation. The sociocultural theory of body image suggests that mass media conveys society’s idealized body standards (Thompson et al., 1999), making weight a sensitive topic for verbal aggression, particularly for adolescents with developing self-concepts. From the perspective of social comparison theory, individuals tend to compare themselves with others in terms of viewpoints and abilities (Festinger, 1954). This framework extends to the body image cognitive comparison model (Nutter et al., 2021), where adolescents engaging in upward appearance comparisons (i.e., comparing themselves to more attractive or socially idealized individuals) (Cohrdes et al., 2021) are prone to experiencing appearance frustration (Lin et al., 2018). This frustration intensifies the fear of negative appearance evaluation (Almenara et al., 2017), ultimately impacting psychological well-being and leading to psychological distress.

### 4.3 Mediating role of social appearance anxiety

The findings suggest that social appearance anxiety partially mediates the relationship between adolescent weight self-stigma and psychological distress, supporting Hypothesis H3. This is consistent with previous research indicating that ‘internalized weight stigma has a strong positive relationship with social appearance anxiety’ (Mustafa et al., 2024) and ‘social appearance anxiety is significantly positively associated with psychological distress’ (Hussain et al., 2023). This is because obese individuals with weight self-stigma are subjected to social exclusion and discrimination because of their weight, which negatively affects their self-esteem and appearance. This, in turn, manifests social appearance anxiety (Mustafa et al., 2024). Social appearance anxiety leads to more social and internal pressures and is highly susceptible to inducing psychological distress (Hussain et al., 2023). Individuals with higher levels of weight self-stigma tend to view their social image negatively and worry about how others perceive them (Radix et al., 2019). These concerns may lead to social anxiety, impaired relationships, career difficulties, and psychological distress (Sahin and Topkaya, 2015). According to the Stigma Hidden Mind Expression Process Model, weight self-stigma from internalizing others’ negative weight evaluations causes adolescents to feel self-devaluation and eager to conceal their stigmatized experiences. However, because weight self-stigma is externally visible and socially threatening, it often triggers negative stress emotions such as social appearance anxiety with diversity and complexity, causing them to experience worry and distress. This emotional burden, combined with social prejudice and discrimination, can significantly increase the likelihood of psychological distress in adolescents.

### 4.4 Chain mediation of fear of negative appearance evaluation and social appearance anxiety

Findings indicated that fear of negative appearance evaluation and social appearance anxiety together mediated the relationship between adolescent weight self-stigma and psychological distress, supporting Hypothesis H4. This is consistent with the idea that ‘social appearance anxiety was strongly associated with threatening information in the situation, which was mainly derived from negative evaluations related to appearance’ (Claes et al., 2012) and ‘the potential mediating role of negative appraisal fear in this relationship between appearance and social anxiety in middle school students’ (Li et al., 2023). There is a significant positive correlation between negative appraisal fear and social anxiety, i.e., the higher the student’s negative appraisal fear, the higher the social anxiety questionnaire score, and vice versa (Iqbal and Ajmal, 2018; Li et al., 2023). Adolescence is a period of intense physical changes and self-concept development, during which concerns about appearance can reach their peak (Pan et al., 2020). When adolescents perceive negative appearance evaluations from others, they may experience fear and panic, leading to greater body dissatisfaction, increased weight concerns, and heightened social appearance anxiety. Additionally, adolescents often exhibit heightened egocentrism, making them feel as though they are constantly being observed and judged by others (Neff, 2003). This perception amplifies their fear of negative evaluation, reinforcing social appearance anxiety and psychological distress. In summary, fear of negative appearance evaluation and social appearance anxiety function as sequential mediators, linking weight self-stigma to psychological distress in adolescents.

## 5 Limitations and implications

This study has several limitations. First, this study selected adolescents from certain regions of Hunan Province as the survey subjects, which may limit the representativeness of the survey results or lead to bias in the results. Future studies should further expand the sample size to enhance the applicability and reliability of the results. Second, this study is missing a discussion of other variables, such as the presence of eating disorders, mood disorders, or bullying experiences among those with weight self-stigma, developmental differences between early and late adolescence (12–18) were also not considered, Future studies could consider more deeply whether individuals are affected by other variables and add comparisons across age groups. In addition, this study used a cross-sectional design based on self-reported questionnaires, while statistical analyses revealed associations between variables, the cross-sectional nature of the study prevents causal inferences regarding the relationships between weight self-stigma, fear of negative appearance evaluation, social appearance anxiety, and psychological distress, in addition to the weight self-stigma questionnaire used in this study, another measurement tool developed by Durso, the Weight Bias Internalization Scale (WBIS) (Durso and Latner, 2008), can also be used to measure weight self-stigma. To further determine the directional, causal nature of these associations as well as to improve the reliability of the data that future studies could use multiple measurement tools or adopt an experimental approach to further explore stable causal relationships between variables. and consider the possibility of adding comparisons between age groups.

Despite these limitations, this study provides novel insights into the relationship between weight self-stigma, fear of negative appearance evaluation, social appearance anxiety, and psychological distress in adolescents. Using a chain-mediated model offers an empirical framework for understanding the mechanisms underlying the association between weight self-stigma and psychological distress. From a practical perspective, these findings can inform intervention strategies aimed at reducing weight self-stigma among adolescents. For schools, it is important to formulate and enforce school rules that explicitly prohibit bullying, ridicule and discrimination based on body weight or physical appearance. Classes can systematically introduce content on weight stigma in ideology and morality classes, psychology classes or class meetings, explaining the harms of weight stigmatization, as well as how to identify and counteract it, and encouraging student associations to carry out activities to promote body acceptance and respect for differences in school activities, publicity materials and school magazines. For individual adolescents, it is important to enhance body image literacy and self-acceptance, learn to differentiate between socio-culturally shaped “ideal bodies” and healthy diversity, and communicate about weight-related concerns with understanding and supportive family members and friends, as well as look for role models of different body types who have a positive body image and healthy lifestyles. By addressing weight self-stigma, such interventions may help prevent adolescents from experiencing psychological distress problems and ensure normal learning and life for adolescents.

## 6 Conclusion

This study found significant correlations between adolescent weight self-stigma, fear of negative appearance evaluation, social appearance anxiety, and psychological distress. In particular, weight self-stigma directly and positively predicted psychological distress. Moreover, fear of negative appearance evaluation partially mediated the relationship between adolescent weight self-stigma and psychological distress, and social appearance anxiety partially mediated the relationship between adolescent weight self-stigma and psychological distress. Additionally, these two factors acted as chain mediators between adolescent weight self-stigma and psychological distress. These findings suggest that effective intervention strategies, such as physical activity and dietary management, should be developed to reduce weight self-stigma. As weight self-stigma decreases, psychological distress in adolescents is also likely to decline, contributing to their physical and mental health development.

## Data Availability

The original contributions presented in the study are included in the article/supplementary material, further inquiries can be directed to the corresponding author.

## References

[ref1] Ahadzadeh A. S. Rafik-Galea S. Alavi M. Amini M. (2018). Relationship between body mass index, body image, and fear of negative evaluation: moderating role of self-esteem. Health Psychol. Open 5:2055102918774251. doi: 10.1177/2055102918774251, PMID: 29977587 PMC6024295

[ref2] Ahorsu D. K. Lin C. Y. Imani V. Griffiths M. D. Su J. A. Latner J. D.. (2020). A prospective study on the link between weight-related self-stigma and binge eating: role of food addiction and psychological distress. Int. J. Eat. Disord. 53, 442–450. doi: 10.1002/eat.23219, PMID: 31905249

[ref3] Ali A. A. Aqeel A. A. Shami M. O. Khodari B. H. Alqassim A. Y. Alessa A. M.. (2024). Relationship between depression, anxiety, stress, and weight self-stigma among youths in Saudi arabia: a nationwide study. Cureus 16:4125. doi: 10.7759/cureus.54125, PMID: 38487156 PMC10939164

[ref4] Almenara C. A. Aimé A. Maïano C. Ejova A. Guèvremont G. Bournival C.. (2017). Weight stigmatization and disordered eating in obese women: the mediating effects of self-esteem and fear of negative appearance evaluation. Eur. Rev. Appl. Psychol. 67, 155–162. doi: 10.1016/j.erap.2017.02.004

[ref5] Andreyeva T. Puhl R. M. Brownell K. D. (2008). Changes in perceived weight discrimination among Americans, 1995–1996 through 2004–2006. Obesity 16, 1129–1134. doi: 10.1038/oby.2008.35, PMID: 18356847

[ref6] Annis N. M. Cash T. F. Hrabosky J. I. (2004). Body image and psychosocial differences among stable average weight, currently overweight, and formerly overweight women: the role of stigmatizing experiences. Body Image 1, 155–167. doi: 10.1016/j.bodyim.2003.12.001, PMID: 18089148

[ref8] Berg N. J. Kiviruusu O. H. Lintonen T. P. Huurre T. M. (2019). Longitudinal prospective associations between psychological symptoms and heavy episodic drinking from adolescence to midlife. Scand. J. Public Health 47, 420–427. doi: 10.1177/1403494818769174, PMID: 29644935

[ref9] Beutel M. E. Klein E. M. Brähler E. Reiner I. Jünger C. Michal M.. (2017). Loneliness in the general population: prevalence, determinants and relations to mental health. BMC Psychiatry 17, 1–7. doi: 10.1186/s12888-017-1262-x, PMID: 28320380 PMC5359916

[ref10] Bharat S. Aggleton P. Tyrer P. (2001). India: HIV and AIDS-related discrimination, stigmatization and denial. Geneva, Switzerland: UNAIDS.

[ref11] Bilge F. Kelecioğlu H. (2008). Psychometric properties of the brief fear of negative evaluation scale: Turkish form. Eurasian J. Educ. Res. 32, 21–38.

[ref12] Boersma K. E. Jarry J. L. (2013). The paradoxical moderating effect of body image investment on the impact of weight-based derogatory media. Body Image 10, 200–209. doi: 10.1016/j.bodyim.2012.11.002, PMID: 23312114

[ref13] Button K. S. Kounali D. Stapinski L. Rapee R. M. Lewis G. Munafò M. R. (2015). Fear of negative evaluation biases social evaluation inference: evidence from a probabilistic learning task. PLoS One 10:e0119456. doi: 10.1371/journal.pone.0119456, PMID: 25853835 PMC4390305

[ref14] Çetin B. Doğan T. Sapmaz F. (2010). Olumsuz değerlendirilme korkusu ölçeği kısa formu’nun Türkçe uyarlaması: Geçerlik ve güvenirlik çalışması. Eğitim ve Bilim 35, 205–216. Available online at: https://educationandscience.ted.org.tr/article/view/871

[ref15] Chan K. L. Lee C. S. C. Cheng C. M. Hui L. Y. So W. T. Yu T. S.. (2019). Investigating the relationship between weight-related self-stigma and mental health for overweight/obese children in Hong Kong. J. Nerv. Ment. Dis. 207, 637–641. doi: 10.1097/NMD.0000000000001021, PMID: 31283726

[ref16] Claes L. Hart T. A. Smits D. Van den Eynde F. Mueller A. Mitchell J. E. (2012). Validation of the social appearance anxiety scale in female eating disorder patients. Eur. Eat. Disord. Rev. 20, 406–409. doi: 10.1002/erv.1147, PMID: 21805536

[ref17] Cohrdes C. Santos-Hövener C. Kajikhina K. Hölling H. (2021). The role of weight-and appearance-related discrimination on eating disorder symptoms among adolescents and emerging adults. BMC Public Health 21, 1–14. doi: 10.1186/s12889-021-11756-y, PMID: 34565379 PMC8474924

[ref18] Coles M. E. Turk C. L. Heimberg R. G. Fresco D. M. (2001). Effects of varying levels of anxiety within social situations: relationship to memory perspective and attributions in social phobia. Behav. Res. Ther. 39, 651–665. doi: 10.1016/S0005-7967(00)00035-8, PMID: 11400710

[ref19] Costello E. J. Copeland W. Angold A. (2011). Trends in psychopathology across the adolescent years: what changes when children become adolescents, and when adolescents become adults? J. Child Psychol. Psychiatry 52, 1015–1025. doi: 10.1111/j.1469-7610.2011.02446.x, PMID: 21815892 PMC3204367

[ref20] Curll S. L. Brown P. M. (2020). Weight stigma and psychological distress: a moderated mediation model of social identification and internalised bias. Body Image 35, 207–216. doi: 10.1016/j.bodyim.2020.09.006, PMID: 33049458

[ref21] Dandan T. Zhonglin W. (2020). Statistical approaches for testing common method bias: problems and suggestions. J. Psychol. Sci. 43, 215–223. doi: 10.16719/j.cnki.1671-6981.20200130

[ref22] Dapeng Z. Wen Z. (2020). Influence, mechanism and intervention of weight stigma on mentaland behavior of overweight and obesity. J. Wuhan Inst. Phys. Educ. 54, 69–74. doi: 10.15930/j.cnki.wtxb.2020.12.010

[ref23] Doherty D. T. Moran R. Kartalova-O'Doherty Y. (2008). Psychological distress, mental health problems and use of health services in Ireland. Dublin: Health Research Board.

[ref24] Doran D. (2011). Psychological distress as a nurse-sensitive outcome. Doran DM nursing outcomes the state of the art. 2nd Edn. Sudbury USA: Jones & Bartlett Learning.

[ref25] Drapeau A. Marchand A. Beaulieu-Prévost D. (2012). “Epidemiology of psychological distress” in Mental illnesses-understanding, prediction and control. ed. LAbate P. L., vol. 69 (London: IntechOpen), 105–106.

[ref26] Durso L. E. Latner J. D. (2008). Understanding self-directed stigma: development of the weight bias internalization scale. Obesity 16, S80–S86. doi: 10.1038/oby.2008.448, PMID: 18978768

[ref7] Edition. Diagnostic and statistical manual of mental disorders. Washington, DC: American Psychiatric Association. (1980) 205–224.

[ref27] Emmer C. Bosnjak M. Mata J. (2020). The association between weight stigma and mental health: a meta-analysis. Obes. Rev. 21:e12935. doi: 10.1111/obr.12935, PMID: 31507062

[ref28] Erath S. A. Flanagan K. S. Bierman K. L. (2008). Early adolescent school adjustment: associations with friendship and peer victimization. Soc. Dev. 17, 853–870. doi: 10.1111/j.1467-9507.2008.00458.x

[ref29] Evans D. L. Charney D. S. Lewis L. Golden R. N. Gorman J. M. Krishnan K. R. R.. (2005). Mood disorders in the medically ill: scientific review and recommendations. Biol. Psychiatry 58, 175–189. doi: 10.1016/j.biopsych.2005.05.001, PMID: 16084838

[ref30] Fagring A. J. Kjellgren K. I. Rosengren A. Lissner L. Manhem K. Welin C. (2008). Depression, anxiety, stress, social interaction and health-related quality of life in men and women with unexplained chest pain. BMC Public Health 8, 1–9. doi: 10.1186/1471-2458-8-165, PMID: 18489751 PMC2416450

[ref31] Faragher E. B. Cass M. Cooper C. L. (2005). The relationship between job satisfaction and health: a meta-analysis. Occup. Environ. Med. 62, 105–112. doi: 10.1136/oem.2002.006734, PMID: 15657192 PMC1740950

[ref32] Farhangi M. A. Emam-Alizadeh M. Hamedi F. Jahangiry L. (2017). Weight self-stigma and its association with quality of life and psychological distress among overweight and obese women. Eat. Weight Disord. 22, 451–456. doi: 10.1007/s40519-016-0288-2, PMID: 27160832

[ref33] Festinger L. (1954). A theory of social comparison processes. Hum. Relat. 7, 117–140. doi: 10.1177/001872675400700202

[ref34] Forbes Y. Donovan C. (2019). The role of internalised weight stigma and self-compassion in the psychological well-being of overweight and obese women. Aust. Psychol. 54, 471–482. doi: 10.1111/ap.12407

[ref35] Gebremedhin H. T. Bifftu B. B. Lebessa M. T. Weldeyes A. Z. Gebru T. T. Petrucka P. (2020). Prevalence and associated factors of psychological distress among secondary school students in Mekelle city, Tigray region, Ethiopia: cross-sectional study. Psychol. Res. Behav. Manage. 13, 473–480. doi: 10.2147/PRBM.S252779 PMC7250291 32547269

[ref36] Geisler M. Berthelsen H. Hakanen J. J. (2019). No job demand is an island–interaction effects between emotional demands and other types of job demands. Front. Psychol. 10:873. doi: 10.3389/fpsyg.2019.00873, PMID: 31057472 PMC6482217

[ref37] Goffman E. (1963). Stigma: notes on the management of spoiled identity. Am. J. Sociol. 2, 2–52.

[ref38] Grant B. F. Stinson F. S. Dawson D. A. Chou S. P. Dufour M. C. Compton W.. (2004). Prevalence and co-occurrence of substance use disorders and independentmood and anxiety disorders: results from the national epidemiologic survey on alcohol and relatedconditions. Arch. Gen. Psychiatry 61, 807–816. doi: 10.1001/archpsyc.61.8.807 15289279

[ref39] Haftgoli N. Favrat B. Verdon F. Vaucher P. Bischoff T. Burnand B.. (2010). Patients presenting with somatic complaints in general practice: depression, anxiety and somatoform disorders are frequent and associated with psychosocial stressors. BMC Fam. Pract. 11, 1–8. doi: 10.1186/1471-2296-11-67, PMID: 20843358 PMC2945969

[ref40] Haikal M. Hong R. Y. (2010). The effects of social evaluation and looming threat on self-attentional biases and social anxiety. J. Anxiety Disord. 24, 345–352. doi: 10.1016/j.janxdis.2010.01.007, PMID: 20176459

[ref41] Harper B. Tiggemann M. (2008). The effect of thin ideal media images on women’s self-objectification, mood, and body image. Sex Roles 58, 649–657. doi: 10.1007/s11199-007-9379-x

[ref42] Hart T. A. Flora D. B. Palyo S. A. Fresco D. M. Holle C. Heimberg R. G. (2008). Development and examination of the social appearance anxiety scale. Assessment 15, 48–59. doi: 10.1177/1073191107306673, PMID: 18258731

[ref43] Hayes A. F. (2013). Introduction to mediation, moderation, and conditional process analysis 51, 335–337.

[ref44] Hayward L. E. Vartanian L. R. Pinkus R. T. (2018). Weight stigma predicts poorer psychological well-being through internalized weight bias and maladaptive coping responses. Obesity 26, 755–761. doi: 10.1002/oby.22126, PMID: 29427370

[ref45] Hazen E. Schlozman S. Beresin E. (2008). Adolescent psychological development: a review. Pediatr. Rev. 29, 161–168. doi: 10.1542/pir.29-5-161, PMID: 18450837

[ref46] Heimberg R. G. (1995). Social phobia: diagnosis, assessment, and treatment. New York: Guilford Press.

[ref47] Henningsen P. Zimmermann T. Sattel H. (2003). Medically unexplained physical symptoms, anxiety, and depression: a meta-analytic review. Psychosom. Med. 65, 528–533. doi: 10.1097/01.PSY.0000075977.90337.E7, PMID: 12883101

[ref48] Himmelstein M. S. Puhl R. M. Quinn D. M. (2017). Intersectionality: an understudied framework for addressing weight stigma. Am. J. Prev. Med. 53, 421–431. doi: 10.1016/j.amepre.2017.04.003, PMID: 28579331

[ref49] Horenstein A. Kaplan S. C. Butler R. M. Heimberg R. G. (2021). Social anxiety moderates the relationship between body mass index and motivation to avoid exercise. Body Image 36, 185–192. doi: 10.1016/j.bodyim.2020.11.010, PMID: 33360475

[ref50] Hussain M. Tariq M. Hussain M. (2023). Social appearance anxiety, psychological distress and quality of life among patients with burn injuries. J. Prof. Appl. Psychol. 4, 418–428. doi: 10.52053/jpap.v4i3.196

[ref51] Iqbal A. Ajmal A. (2018). Fear of negative evaluation and social anxiety in young adults. Peshawar J. Psychol. Behav. Sci. 4, 45–53. doi: 10.32879/picp.2018.4.1.45

[ref52] Jackson S. E. Steptoe A. Beeken R. J. Croker H. Wardle J. (2015). Perceived weight discrimination in England: a population-based study of adults aged ⩾ 50 years. Int. J. Obes. 39, 858–864. doi: 10.1038/ijo.2014.186, PMID: 25327975 PMC4309989

[ref53] Kendler K. S. Myers J. Prescott C. A. (2005). Sex differences in the relationship between social support and risk for major depression: a longitudinal study of opposite-sex twin pairs. Am. J. Psychiatry 162, 250–256. doi: 10.1176/appi.ajp.162.2.250, PMID: 15677587

[ref54] Kessler R. C. Barker P. R. Colpe L. J. Epstein J. F. Gfroerer J. C. Hiripi E.. (2003). Screening for serious mental illness in the general population. Arch. Gen. Psychiatry 60, 184–189. doi: 10.1001/archpsyc.60.2.184, PMID: 12578436

[ref55] Kornienko O. Santos C. E. (2014). The effects of friendship network popularity on depressive symptoms during early adolescence: moderation by fear of negative evaluation and gender. J. Youth Adolesc. 43, 541–553. doi: 10.1007/s10964-013-9979-4, PMID: 23832253

[ref56] Koskina A. Van den Eynde F. Meisel S. Campbell I. Schmidt U. (2011). Social appearance anxiety and bulimia nervosa. Eat. Weight Disord. 16, e142–e145. doi: 10.1007/BF03325321 21989100

[ref57] Kroenke K. (2003). Patients presenting with somatic complaints: epidemiology, psychiatric co-morbidity and management. Int. J. Methods Psychiatr. Res. 12, 34–43. doi: 10.1002/mpr.140, PMID: 12830308 PMC6878426

[ref58] Kroenke K. Outcalt S. Krebs E. Bair M. J. Wu J. Chumbler N.. (2013). Association between anxiety, health-related quality of life and functional impairment in primary care patients with chronic pain. Gen. Hosp. Psychiatry 35, 359–365. doi: 10.1016/j.genhosppsych.2013.03.020, PMID: 23639186

[ref59] Latner J. D. Barile J. P. Durso L. E. O'Brien K. S. (2014). Weight and health-related quality of life: the moderating role of weight discrimination and internalized weight bias. Eat. Behav. 15, 586–590. doi: 10.1016/j.eatbeh.2014.08.014, PMID: 25215477

[ref60] Lee H. Lee E. Y. Greene B. Shin Y.-j. (2019). Psychological distress among adolescents in Laos, Mongolia, Nepal, and Sri Lanka. Asian Nurs. Res. 13, 147–153. doi: 10.1016/j.anr.2019.04.001, PMID: 31003005

[ref61] Li Y. (2020). Linking body esteem to eating disorders among adolescents: a moderated mediation model. J. Health Psychol. 25, 1755–1770. doi: 10.1177/1359105319886048, PMID: 31674200

[ref62] Li J. Jia S. Wang L. Zhang M. Chen S. (2023). Relationships among inferiority feelings, fear of negative evaluation, and social anxiety in Chinese junior high school students. Front. Psychol. 13:1015477. doi: 10.3389/fpsyg.2022.1015477, PMID: 36704691 PMC9872515

[ref63] Li J. Wang H. Li M. Shen Q. Li X. Zhang Y.. (2020). Effect of alcohol use disorders and alcohol intake on the risk of subsequent depressive symptoms: a systematic review and meta-analysis of cohort studies. Addiction 115, 1224–1243. doi: 10.1111/add.14935, PMID: 31837230

[ref64] Lillis J. Luoma J. B. Levin M. E. Hayes S. C. (2010). Measuring weight self-stigma: the weight self-stigma questionnaire. Obesity 18, 971–976. doi: 10.1038/oby.2009.353, PMID: 19834462

[ref65] Lin Y. C. Latner J. D. Fung X. C. Lin C. Y. (2018). Poor health and experiences of being bullied in adolescents: self-perceived overweight and frustration with appearance matter. Obesity 26, 397–404. doi: 10.1002/oby.22041, PMID: 29090855

[ref66] Lin C.-Y. Tsai M.-C. Liu C.-H. Lin Y.-C. Hsieh Y.-P. Strong C. (2019). Psychological pathway from obesity-related stigma to anxiety via internalized stigma and self-esteem among adolescents in Taiwan. Int. J. Environ. Res. Public Health 16:4410. doi: 10.3390/ijerph16224410, PMID: 31718003 PMC6887789

[ref67] Liu H. Zhang M. Yang Q. Yu B. (2020). Gender differences in the influence of social isolation and loneliness on depressive symptoms in college students: a longitudinal study. Soc. Psychiatry Psychiatr. Epidemiol. 55, 251–257. doi: 10.1007/s00127-019-01726-6, PMID: 31115597

[ref68] Lovibond S. Lovibond P. (1995). Depression anxiety stress scale-21 (DASS-21), vol. 10. Sydney: School of Psychology, University of New South Wales, 13.

[ref69] Löwe B. Spitzer R. L. Williams J. B. Mussell M. Schellberg D. Kroenke K. (2008). Depression, anxiety and somatization in primary care: syndrome overlap and functional impairment. Gen. Hosp. Psychiatry 30, 191–199. doi: 10.1016/j.genhosppsych.2008.01.001, PMID: 18433651

[ref70] Lucibello K. M. Nesbitt A. E. Solomon-Krakus S. Sabiston C. M. (2021). Internalized weight stigma and the relationship between weight perception and negative body-related self-conscious emotions. Body Image 37, 84–88. doi: 10.1016/j.bodyim.2021.01.010, PMID: 33596497

[ref71] Lundgren J. D. Anderson D. A. Thompson J. K. (2004). Fear of negative appearance evaluation: development and evaluation of a new construct for risk factor work in the field of eating disorders. Eat. Behav. 5, 75–84. doi: 10.1016/S1471-0153(03)00055-2, PMID: 15000956

[ref72] Major B. O’brien L. T. (2005). The social psychology of stigma. Annu. Rev. Psychol. 56, 393–421. doi: 10.1146/annurev.psych.56.091103.070137, PMID: 15709941

[ref73] Mannan M. Mamun A. Doi S. Clavarino A. (2016). Prospective associations between depression and obesity for adolescent males and females-a systematic review and meta-analysis of longitudinal studies. PLoS One 11:e0157240. doi: 10.1371/journal.pone.0157240, PMID: 27285386 PMC4902254

[ref74] Marchand A. Demers A. Durand P. (2005). Do occupation and work conditions really matter? A longitudinal analysis of psychological distress experiences among Canadian workers. Sociol. Health Illn. 27, 602–627. doi: 10.1111/j.1467-9566.2005.00458.x, PMID: 16078903

[ref75] Marchand A. Drapeau A. Beaulieu-Prévost D. (2012). Psychological distress in Canada: the role of employment and reasons of non-employment. Int. J. Soc. Psychiatry 58, 596–604. doi: 10.1177/0020764011418404, PMID: 21873292 PMC3654934

[ref76] McCabe R. E. Antony M. M. Summerfeldt L. J. Liss A. Swinson R. P. (2003). Preliminary examination of the relationship between anxiety disorders in adults and self-reported history of teasing or bullying experiences. Cogn. Behav. Ther. 32, 187–193. doi: 10.1080/16506070310005051, PMID: 16291550

[ref77] Munir M. Dawood S. (2021). Weight stigma and disordered eating behaviors in Pakistani overweight adolescents: the mediating role of body esteem. Eat. Weight Disord. 26, 1939–1948. doi: 10.1007/s40519-020-01038-8, PMID: 33068276

[ref78] Mustafa K. Erkan F.M. Gültekin A. Arslan M. Budak F.K. (2024). The correlation of weight self-stigma and social appearance anxiety in people with obesity. American, research square.

[ref79] Myre M. Glenn N. M. Berry T. R. (2021). Exploring the impact of physical activity-related weight stigma among women with self-identified obesity. Qual. Res. Sport Exerc. Health 13, 586–603. doi: 10.1080/2159676X.2020.1751690

[ref80] Neff K. (2003). Self-compassion: an alternative conceptualization of a healthy attitude toward oneself. Self Identity 2, 85–101. doi: 10.1080/15298860309032

[ref81] Nichelson K. D. (2020). Predictors of healthcare avoidance: an examination of weight self-stigma and anticipated weight identity threat: Spalding University.

[ref82] Nonterah C. W. Hahn N. C. Utsey S. O. Hook J. N. Abrams J. A. Hubbard R. R.. (2015). Fear of negative evaluation as a mediator of the relation between academic stress, anxiety and depression in a sample of Ghanaian college students. Psychol. Dev. Soc. 27, 125–142. doi: 10.1177/0971333614564747

[ref83] Nutter S. Russell-Mayhew S. Saunders J. F. (2021). Towards a sociocultural model of weight stigma. Eat. Weight Disord. 26, 999–1005. doi: 10.1007/s40519-020-00931-6, PMID: 32462360

[ref84] O'Brien K. S. Latner J. D. Puhl R. M. Vartanian L. R. Giles C. Griva K.. (2016). The relationship between weight stigma and eating behavior is explained by weight bias internalization and psychological distress. Appetite 102, 70–76. doi: 10.1016/j.appet.2016.02.032, PMID: 26898319

[ref85] Özdemir A. A. Türkben H. (2023). The relationship between weight self-stigma, depression and loneliness in people with obesity. Afr. Health Sci. 23, 696–704. doi: 10.4314/ahs.v23i3.80, PMID: 38357107 PMC10862624

[ref86] Pan S. Yufei H. Hanzhi Z. Xuechen L. Hong C. (2020). Negative effect of negative body image on adolescents. Adv. Psychol. Sci. 28, 294–304. doi: 10.3724/SP.J.1042.2020.00294

[ref87] Papadopoulos S. Brennan L. (2015). Correlates of weight stigma in adults with overweight and obesity: a systematic literature review. Obesity 23, 1743–1760. doi: 10.1002/oby.21187, PMID: 26260279

[ref88] Phelps N. H. Singleton R. K. Zhou B. Heap R. A. Mishra A. Bennett J. E.. (2024). Worldwide trends in underweight and obesity from 1990 to 2022: a pooled analysis of 3663 population-representative studies with 222 million children, adolescents, and adults. Lancet 403, 1027–1050. doi: 10.1016/S0140-6736(23)02750-2, PMID: 38432237 PMC7615769

[ref89] Pont S. J. Puhl R. Cook S. R. Slusser W. (2017). Stigma experienced by children and adolescents with obesity. Pediatrics 140:3034. doi: 10.1542/peds.2017-3034, PMID: 29158228

[ref90] Prunty A. Clark M. K. Hahn A. Edmonds S. O’Shea A. (2020). Enacted weight stigma and weight self stigma prevalence among 3821 adults. Obes. Res. Clin. Pract. 14, 421–427. doi: 10.1016/j.orcp.2020.09.003, PMID: 32952068

[ref91] Puhl R. M. King K. M. (2013). Weight discrimination and bullying. Best Pract. Res. Clin. Endocrinol. Metab. 27, 117–127. doi: 10.1016/j.beem.2012.12.002, PMID: 23731874

[ref92] Radix A. K. Rinck M. Becker E. S. Legenbauer T. (2019). The mediating effect of specific social anxiety facets on body checking and avoidance. Front. Psychol. 9:2661. doi: 10.3389/fpsyg.2018.02661, PMID: 30671002 PMC6331477

[ref93] Ranjit A. Korhonen T. Buchwald J. Heikkilä K. Tuulio-Henriksson A. Rose R. J.. (2019). Testing the reciprocal association between smoking and depressive symptoms from adolescence to adulthood: a longitudinal twin study. Drug Alcohol Depend. 200, 64–70. doi: 10.1016/j.drugalcdep.2019.03.012, PMID: 31100637

[ref94] Rapee R. M. Heimberg R. G. (1997). A cognitive-behavioral model of anxiety in social phobia. Behav. Res. Ther. 35, 741–756. doi: 10.1016/S0005-7967(97)00022-3, PMID: 9256517

[ref95] Ridner S. H. (2004). Psychological distress: concept analysis. J. Adv. Nurs. 45, 536–545. doi: 10.1046/j.1365-2648.2003.02938.x, PMID: 15009358

[ref96] Rog J. Nowak K. Wingralek Z. (2024). The relationship between psychological stress and anthropometric, biological outcomes: a systematic review. Medicina 60:1253. doi: 10.3390/medicina60081253, PMID: 39202534 PMC11356149

[ref97] Roth D. A. Coles M. E. Heimberg R. G. (2002). The relationship between memories for childhood teasing and anxiety and depression in adulthood. J. Anxiety Disord. 16, 149–164. doi: 10.1016/S0887-6185(01)00096-2, PMID: 12194541

[ref98] Rubino F. Puhl R. M. Cummings D. E. Eckel R. H. Ryan D. H. Mechanick J. I.. (2020). Joint international consensus statement for ending stigma of obesity. Nat. Med. 26, 485–497. doi: 10.1038/s41591-020-0803-x, PMID: 32127716 PMC7154011

[ref99] Sahin E. Topkaya N. (2015). Factor structure of the social appearance anxiety scale in Turkish early adolescents. Univ. J. Educ. Res. 3, 513–519. doi: 10.13189/ujer.2015.030806

[ref100] Schram S. J. Olson K. L. Panza E. Lillis J. (2024). The impact of weight self-stigma on weight-loss treatment engagement and outcome. Obes. Sci. Pract. 10:e70015. doi: 10.1002/osp4.70015, PMID: 39483439 PMC11523143

[ref101] Shepherd L. Reynolds D. P. Turner A. O’Boyle C. P. Thompson A. R. (2019). The role of psychological flexibility in appearance anxiety in people who have experienced a visible burn injury. Burns 45, 942–949. doi: 10.1016/j.burns.2018.11.015, PMID: 30591252

[ref102] Stevens S. D. Herbozo S. Martinez S. N. (2018). Weight stigma, depression, and negative appearance commentary: exploring BMI as a moderator. Stigma Health. 3, 108–115. doi: 10.1037/sah0000081

[ref103] Stunkard A. J. Faith M. S. Allison K. C. (2003). Depression and obesity. Biol. Psychiatry 54, 330–337. doi: 10.1016/S0006-3223(03)00608-5, PMID: 12893108

[ref104] Thapar A. Collishaw S. Pine D. S. Thapar A. K. (2012). Depression in adolescence. Lancet 379, 1056–1067. doi: 10.1016/S0140-6736(11)60871-4, PMID: 22305766 PMC3488279

[ref105] Thomas C. Keery H. Williams R. Thompson J. (1998). “The fear of negative appearance evaluation scale: Development and preliminary validation” in Annual meeting of the Association for the Advancement of Behavior Therapy (Washington, DC).

[ref106] Thompson J. K. Heinberg L. J. Altabe M. Tantleef-Dunn S. (1999). “Theory assessment, and treatment of body image disturbance” in Exacting beauty: Theory, assessment, and treatment of body image disturbance. eds. Thomson J. K. Heinberg L. J. Altabe M. N. Tantlee-Dunn (Washington, DC: American Psychological Association).

[ref107] Tomiyama A. J. (2014). Weight stigma is stressful. A review of evidence for the cyclic obesity/weight-based stigma model. Appetite 82, 8–15. doi: 10.1016/j.appet.2014.06.108, PMID: 24997407

[ref108] Uğur E. Kaya Ç. Tanhan A. (2021). Psychological inflexibility mediates the relationship between fear of negative evaluation and psychological vulnerability. Curr. Psychol. 40, 4265–4277. doi: 10.1007/s12144-020-01074-8, PMID: 32982124 PMC7509823

[ref109] Van Oppen P. Smit J. Van Balkom A. Zitman F. Nolen W. Beekman A.. (2007). Comorbidity of anxiety and depression. Eur. Psychiatry 22:S333. doi: 10.1016/j.eurpsy.2007.01.1110 21294994

[ref110] Vartanian L. R. Smyth J. M. (2013). Primum non nocere: obesity stigma and public health. J. Bioethical Inquiry 10, 49–57. doi: 10.1007/s11673-012-9412-9, PMID: 23288439

[ref111] Waasdorp T. E. Mehari K. Bradshaw C. P. (2018). Obese and overweight youth: risk for experiencing bullying victimization and internalizing symptoms. Am. J. Orthopsychiatry 88, 483–491. doi: 10.1037/ort0000294, PMID: 29355366

[ref112] Wan Z. Li S. Fang S. (2024). The effect of negative physical self on social anxiety in college students: the bidirectional chain mediation roles of fear of negative evaluation and regulatory emotional self-efficacy. Psychol. Res. Behav. Manag. 17, 2055–2066. doi: 10.2147/PRBM.S457405, PMID: 38800523 PMC11122180

[ref113] Wen G. Wenzhen S. Lan Z. Rong S. (2023a). The double-edged sword effects of weight self-stigma on adolescent'exercise behavior: an empirical analysis based on the social information processing theory. Chin. Sport Sci. 43, 60–68. doi: 10.16469/j.css.202311007

[ref114] Wen G. Wenzhen S. Yin G. Lan Z. (2024a). Effect of HAES intervention on obese adolescents’eating disorder tendency andphysical exercise behavior: the mediating effect of weight self-stigma. J. Phys. Educ. 31, 62–67. doi: 10.16237/j.cnki.cn44-1404/g8.2024.05.015

[ref115] Wen G. Xiaokang M. Yang L. (2024b). The influence mechanism of weight self-stigma on adolescents’ exercise avoidance: an empirical analysis based on the transactional theory of stress and coping. J. Beijing Sport Univ. 47, 19–32. doi: 10.19582/j.cnki.11-3785/g8.2024.05.002

[ref116] Wen G. Yang L. Xiaokang M. Qiangfeng Z. (2023b). Influencing mechanism of weight self-stigma on adolescents’ home physical activity: an empirical analysis based on basic psychological need theory. J. Wuhan Sport Univ. 57, 62–70. doi: 10.15930/j.cnki.wtxb.2023.09.012

[ref117] Wu Y. K. Berry D. C. (2018). Impact of weight stigma on physiological and psychological health outcomes for overweight and obese adults: a systematic review. J. Adv. Nurs. 74, 1030–1042. doi: 10.1111/jan.13511, PMID: 29171076

[ref118] Zhao C. Dai B. (2016). Relationship of fear of negative evaluation and social anxiety in college students. Chin. J. Health Psychol. 24, 1746–1749. doi: 10.13342/j.cnki.cjhp.2016.11.038

[ref119] Zhong Y. Zhang J. (2011). The mediating effects of fears of evaluation on the relations between self-esteem and social anxiety for college students. Psychol Dev Edu 27, 506–512.

[ref120] Zhonglin W. Baojuan Y. (2014). Analyses of mediating effects: the development of methods and models. Adv. Psychol. Sci. 22, 731–745.

